# Creatinine Trends to Detect Ibuprofen-Related Maturational Adverse Drug Events in Neonatal Life: A Simulation Study for the ELBW Newborn

**DOI:** 10.3389/fphar.2020.610294

**Published:** 2021-01-25

**Authors:** Tamara van Donge, Karel Allegaert, Marc Pfister, Anne Smits, John van den Anker

**Affiliations:** ^1^ Pediatric Pharmacology and Pharmacometrics, University Children’s Hospital Basel (UKBB), University of Basel, Basel, Switzerland; ^2^ Department of Development and Regeneration, KU Leuven, Leuven, Belgium; ^3^ Department of Pharmaceutical and Pharmacological Sciences, KU Leuven, Leuven, Belgium; ^4^ Department of Hospital Pharmacy, Erasmus MC University Medical Center, Rotterdam, Netherlands; ^5^ Neonatal Intensive Care Unit, University Hospitals Leuven, Leuven, Belgium; ^6^ Division of Clinical Pharmacology, Children’s National Health Hospital, Washington, DC, United States; ^7^ Intensive Care and Department of Pediatric Surgery, Erasmus MC Sophia Children’s Hospital, Rotterdam, Netherlands

**Keywords:** serum creatinine, ibuprofen, nephrotoxicity, creatinine clearance, ELBW neonates

## Abstract

**Background:** Recognizing a change in serum creatinine concentrations is useful to detect a renal adverse drug reaction signal. Assessing and characterizing the nephrotoxic side-effects of drugs in extremely low birth weight (ELBW, ≤1000 g) neonates remain challenging due to the high variability in creatinine in this population. This study aims to investigate and quantify the impact of ibuprofen treatment on kidney function, reflected by serum creatinine.

**Method:** A recently developed dynamical model for serum creatinine was used to simulate creatinine profiles for typical, reference ELBW neonates with varying gestational and postnatal ages whilst being exposed to ibuprofen treatment.

**Results:** The increase of serum creatinine concentrations due to ibuprofen treatment is most apparent during the first week of life. The difference in serum creatinine values between ibuprofen-exposed vs. non-exposed neonates decreases with increasing postnatal age, independent of gestational age.

**Conclusion:** The difference in serum creatinine concentrations between ibuprofen-exposed vs. non-exposed neonates decreases with postnatal age, indicating an increased clearing capacity and resulting in a weak ibuprofen-related adverse drug reaction signal beyond early neonatal life.

## Introduction

Extremely low birth weight (ELBW, ≤1,000 g) infants are born during active nephrogenesis, making their kidneys extremely vulnerable to damage by external factors such as exposure to nephrotoxic drugs or diseases such as neonatal sepsis. Although the development of nephrons will continue after preterm delivery, the final amount of nephrons will be less for infants who are born prematurely with an ELBW (Faa et al., 2010; Abitbol et al., 2016; Hoogenboom et al., 2020).

Serum creatinine is commonly used as a surrogate biomarker for glomerular filtration rate as well as to assess kidney injury. Serum creatinine concentrations in preterm neonates are highly variable due to rapid changes in maturation (i.e., developmental physiology) and intercurrent events such as neonatal sepsis or therapeutic interventions for neonatal diseases (Vieux et al., 2010; George et al., 2011; van den Anker et al., 2018; Allegaert et al., 2020). The variations in serum creatinine values in these extreme preterm infants are still not fully understood and the accurate assessment of kidney function remains challenging. Although developed equations such as the (bedside) Schwartz formula support clinicians in estimating the glomerular filtration rate (GFR), the underlying physiology and the impact of pharmacotherapy have not been fully elucidated for ELBW neonates (Schwartz et al., 1976; Schwartz et al., 2009). Recently, the quantitative effect of maturational changes such as gestational age and postnatal age, together with the impact of mode of delivery and ibuprofen treatment on the serum creatinine in ELBW neonates, has been reported (van Donge et al., 2020). This study showed that gestational age was a major determinant of the initial creatinine concentration at birth, suggesting gestational-dependent maternal creatinine transfer until birth (van Donge et al., 2020).

The fundamental aspects of neonatal clinical pharmacology such as exposure to a multitude of drugs, a highly variable population as reflected by the 10-fold difference in birth weight on admission: 0.5–5 kg, and rapid growth and development result in extensive within- and between-subject variability in drug disposition during the first months of life (Allegaert et al., 2008; Allegaert et al., 2014). Ibuprofen, a nonsteroidal anti-inflammatory drug (NSAID), is often prescribed to preterm neonates as pharmacological treatment of a symptomatic patent ductus arteriosus and is known for its nephrotoxic side-effects (Mitra et al., 2018). The nephrotoxic effects of ibuprofen are related to its mechanism of action (i.e., inhibition of cyclooxygenase, resulting in decreased prostaglandin synthesis) (Fanos et al., 2010). The induced prostaglandin inhibition of ibuprofen may result in renal hypoperfusion as prostaglandin E_2_, being synthesized along the nephron, and contributes to the regulation of renal perfusion and GFR by neutralizing the action of vasoconstrictive substances such as angiotensin II (Fanos et al., 2005; Antonucci and Fanos, 2009).

The aim of this study was to assess and quantify the effect of ibuprofen on serum creatinine profiles of ELBW neonates of various postnatal and gestational ages. This knowledge may support the interpretation of serum creatinine values in this neonatal subpopulation and inform clinicians on the kidney function and potential kidney injury in their patients.

## Method

### Ethics

The studies involving human participants were reviewed and approved by the Ethics Committee UZ/KU Leuven (S63405, June 17, 2020). As this was a retrospective analysis of serum creatinine and clinical data, written consent from the legal guardians was not required to participate in this study in accordance with the national legislation and the institutional requirements.

### Serum Creatinine Dynamic Model

We applied a recently published dynamic model that characterized the serum creatinine concentrations for ELBW infants (van Donge et al., 2020). The model was developed based on 4,026 serum creatinine concentrations collected during the first 6 weeks of life in 217 ELBW neonates. The median gestational age and birth weight were 27 weeks (26–28 weeks IQR) and 830 g (720–910 g IQR), respectively. The mode of delivery and gestational age showed an association with postnatal maturation of the creatinine clearance (faster clearance increase with advancing GA and after C-section). In this previous study, the elimination rate of creatinine (ket) was characterized as $ket\;=\;\frac{CL\left(t\right)}{Vd}\;=\;\frac{\left(CL_{BL}+\frac{\mathrm{emax}\;\times \;t^{Hill}}{t_{50}^{Hill}+\;t^{Hill}}\;\right)}{0.7_{(\frac{L}{kg})}\times \;weight_{\left(kg\right)}}$, where *t* reflects postnatal age (days), *CL*_BL_ reflects baseline creatinine clearance (L/day), emax is the maximum additional achieved clearance (L/day), t_50_ corresponds to the time point where half of emax is achieved, and the Hill coefficient describes the steepness of the creatinine-time relationship. Additional details on the dynamic model can be found in the original article (van Donge et al., 2020). Ibuprofen treatment was included in the model on creatinine clearance at baseline (*CL*_BL_) and accounted for a 5% decrease when ibuprofen was administered [*CL*_BL*i*_ = 0.075 L/day × (1 + 2.55)]. The study showed that ibuprofen treatment is associated with a decrease in creatinine clearance, whereas inotropic agents were not associated with changes in serum creatinine or creatinine clearance and therefore not included in the model. This study provided new insights into the perinatal factors (related to physiological and pathophysiological) affecting serum creatinine and therefore influencing kidney function. The current study aims to quantify the impact of ibuprofen treatment on serum creatinine concentrations at various periods during early and late neonatal life, addressing the potential adverse drug reactions of ibuprofen, affected by postnatal maturation.

To illustrate the effect of ibuprofen treatment on serum creatinine profiles in cases with different gestational and postnatal ages, we simulated serum creatinine profiles during ibuprofen exposure for four typical reference neonates with varying gestational ages of 24, 27, 29, and 32 weeks, respectively. Their respective birth weight amounted to 621, 779, 840, and 889 g. The ibuprofen treatment was comprised of three different treatment periods of three consecutive days: either days 1–3 (first week of life), or day 15 to day 17 (third week of life), or day 29 to day 31 (fifth week of life) after birth. BSA at the start of the first treatment period (day 1) amounted to 0.07, 0.08, 0.09, and 0.09 m^2^, for our four typical reference neonates, respectively, and BSA at the end of the third treatment period (day 31) was 0.09, 0.11, 0.11, and 0.12 m^2^. In the current dataset, ibuprofen was administered according to the current label, i.e., 10, 5, and 5 mg/kg, respectively, at 24 h intervals. Simulations were performed using the mlxR package in R (version 3.5.1; R Development Core Team, Vienna, Austria, http://r-project.org), with the inclusion of the population parameters derived of the dynamic model, which was developed in Monolix (version 2019R1. Antony, France: Lixoft SAS, 2020, http://lixoft.com/products/monolix/). We compared the predicted serum creatinine concentration and creatinine clearance over a period of 6 weeks after birth for typical reference ELBW cases who were exposed to ibuprofen or not.

## Results

### Serum Creatinine Dynamic Model

**FIGURE 1:**
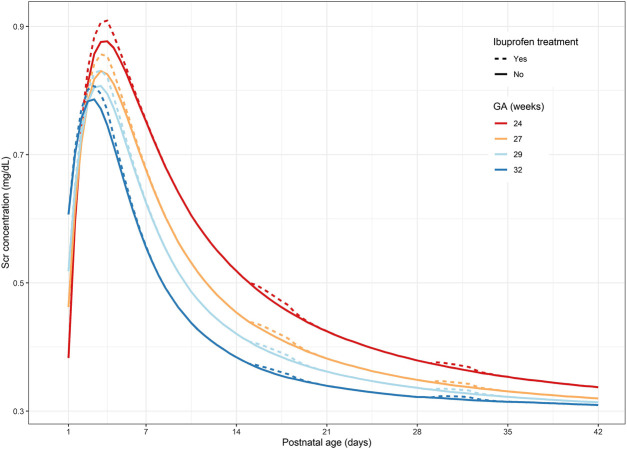
Predicted serum creatinine concentration-time profiles for four typical reference GA ELBW neonates: dotted lines represent serum creatinine concentration under ibuprofen treatment; solid line represents the absence of ibuprofen treatment. GA, gestational age; ELBW, extremely low birth weight.

Predicted serum creatinine concentration profiles for four typical reference ELBW neonates are shown in Figure 1. Generally, an increase in serum creatinine concentrations is observed during treatment with ibuprofen, with a more pronounced effect during the early neonatal life, i.e., the first week of life. The difference in the serum creatinine concentrations between patients receiving ibuprofen and non-exposed patients decreases with postnatal age; this trend is independent of gestational age (Table 1). Table 2 illustrates the serum creatinine concentrations for the four different gestational ages, separated by the presence or absence of ibuprofen treatment for the three different postnatal periods (week 1, 3, or 5 after birth).

**TABLE 1:** Differences in serum creatinine concentration (mg/dl) calculated at the end of the treatment period for three ibuprofen treatment periods starting at different postnatal ages and separated per gestational age group.

	Differences in creatinine concentration (mg/dl)		
Gestational age	Period 1 (days 1–3)	Period 2(days 15–17)	Period 3(days 29–31)
24 weeks	0.030	0.010	0.006
27 weeks	0.026	0.008	0.005
29 weeks	0.025	0.007	0.005
32 weeks	0.023	0.006	0.004

**TABLE 2:** Serum creatinine concentrations (mg/dl) for different gestational ages, exposed to ibuprofen treatment or without ibuprofen treatment. Separated for three treatment periods, starting at day 1, day 15, and day 29 of postnatal life and lasting for 3 days.

	Creatinine concentration (mg/dl)					
	Period 1 (days 1–3)		Period 2 (days 15–17)		Period 3 (days 29–31)	
	Before	After	Before	After	Before	After
Gestational age of 24 weeks						
Ibuprofen	0.383	0.906	0.500	0.472	0.375	0.371
No ibuprofen	0.383	0.876	0.500	0.462	0.375	0.365
Gestational age of 27 weeks						
Ibuprofen	0.462	0.857	0.439	0.419	0.345	0.344
No ibuprofen	0.462	0.830	0.439	0.411	0.345	0.339
Gestational age of 29 weeks						
Ibuprofen	0.518	0.832	0.408	0.391	0.334	0.333
No ibuprofen	0.518	0.807	0.408	0.384	0.334	0.328
Gestational age of 32 weeks						
Ibuprofen	0.607	0.795	0.374	0.360	0.321	0.322
No ibuprofen	0.607	0.772	0.374	0.355	0.321	0.318

The extent of the increase of serum creatinine concentrations due to ibuprofen treatment is the most pronounced during the first week of life (i.e., round 1). For instance, for a neonate born at 24 weeks of gestation, the creatinine concentrations increase from 0.383 to 0.906 or 0.876 mg/dl in the presence or absence of ibuprofen treatment, respectively (Table 2). This elevation of creatinine concentrations is less pronounced later in life, as the difference in creatinine concentrations between ibuprofen treatment and the baseline is practically negligible for weeks 3 and 5 after birth (Table 1).

**FIGURE 2:**
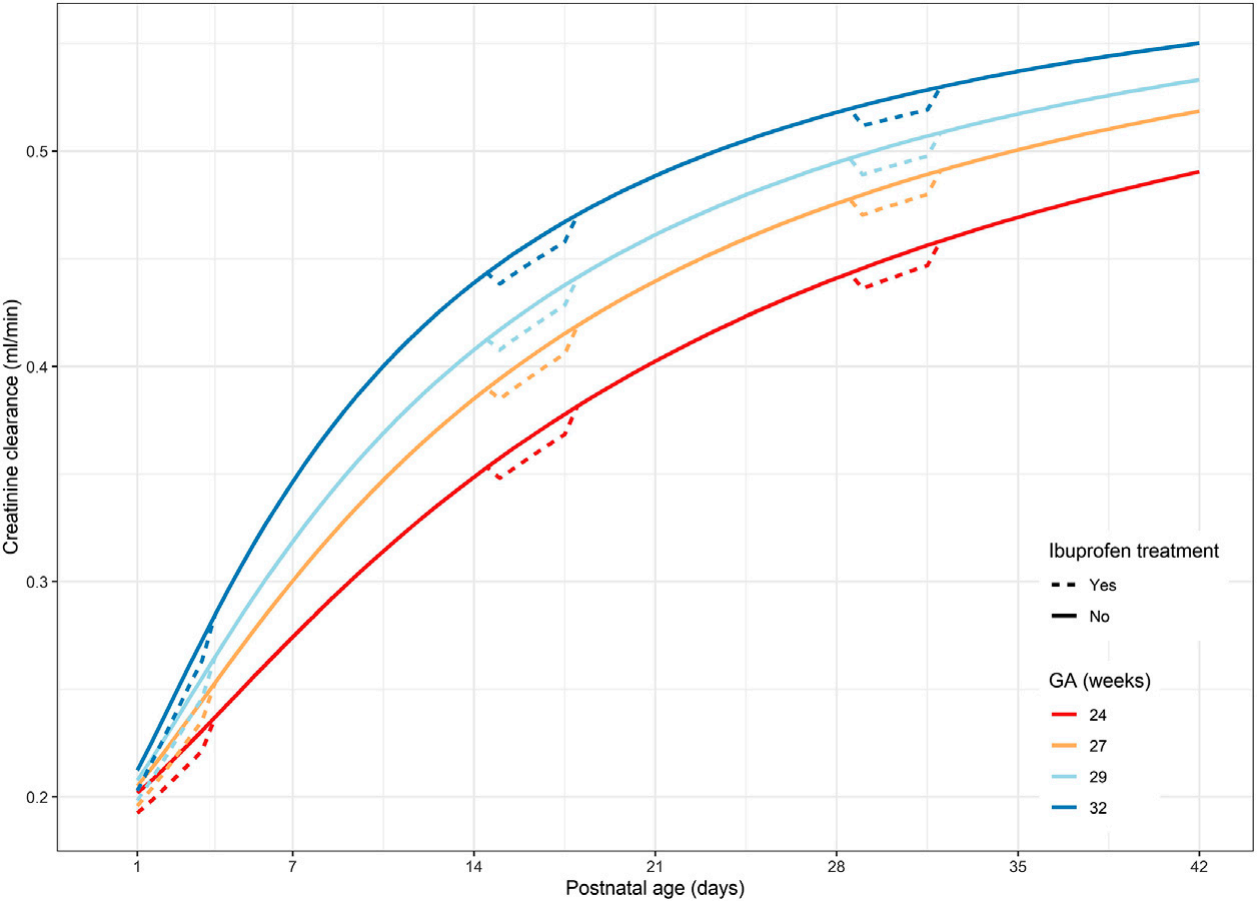
Predicted creatinine clearance profiles for four typical reference GA ELBW neonates: dotted lines represent creatinine clearance under ibuprofen treatment; solid line represents the absence of ibuprofen treatment. GA, gestational age; ELBW, extremely low birth weight.

Focusing on the different gestational ages, the initial increase in serum creatinine concentrations as observed during the 5 days of life is most profound in the more immature neonates. This elevation of creatinine concentrations is the highest in extremely premature neonates (an increase of 0.03 mg/dl for a 24-week-old neonate) and decreases with gestational age (an increase of 0.02 mg/dl for a 32-week-old neonate, Table 1). Figure 2 illustrates the increasing clearance capacities during the postnatal period for ELBW neonates, depending on gestational age. Creatinine clearance is reduced when the neonate is exposed to ibuprofen, but this decrease is proportionally much more relevant in the first week of postnatal life. The reduction in creatinine clearance is assumed to be *absolute* (−0.0094 ml/min), indicating no difference between gestational and postnatal age. The reduction in creatinine clearance due to ibuprofen treatment is therefore *proportionally higher* in the early days after birth (−5%), when clearance capacity is much limited, as compared to week 5 after birth (approximately −2%).

## Discussion

This simulation study illustrated that the effect of ibuprofen treatment is most apparent during the first week of life, independent of gestational age. This difference in serum creatinine concentrations decreases with postnatal age, illustrating the increased renal clearance capacity during the early weeks of life and simultaneous blunting of the ibuprofen-related adverse drug reaction signal.

During the first days after birth, many physiological, maturational, and potential pathological processes occur. The increase in serum creatinine concentrations during the first days after birth is the net phenotypic result of limited clearance capacities, drug exposure, tubular reabsorption (due to leaky tubules), maternal creatinine transfer, and/or neonatal comorbidities. It remains challenging to disentangle these contributing components.

A previous study showed that the half-life of amikacin in preterm neonates with a gestational age less than 31 weeks, who received either ibuprofen or placebo, was significantly higher in the patients who received ibuprofen (16.4 vs. 14.2 h) in early neonatal life (Allegaert et al., 2004). Similar results were also observed for amikacin clearance (0.36 vs. 0.6 ml/kg/min), indicating that ibuprofen reduces the clearance of amikacin. Being aware of the fact that amikacin is eliminated completely by glomerular filtration, these findings can also be applied to the clearance of creatinine (Allegaert et al., 2004). It has to be acknowledged that these data were generated in the first 3 days of postnatal age (<72 h after birth); therefore, the magnitude of this negative effect of ibuprofen exposure to the glomerular filtration cannot simply be extrapolated beyond this age. The current study confirmed that ibuprofen has a more pronounced effect on the serum creatinine concentrations (and GFR) when exposed during the first week of life as compared to later in neonatal life.

Vieux et al. illustrated that, among all drugs that are potentially nephrotoxic in very preterm infants, ibuprofen alone proved nephrotoxic (decrease in estimated GFR) at one-month follow-up (Vieux et al., 2011). Besides the type of drug, the amount of administered drugs are as well of relevance; it has been shown that very low birth weight infants with acute kidney injury received more nephrotoxic drugs than those who did not, as illustrated by Rhone et al. (2014). In contrast, long-term outcome data on renal function in young adolescence provided evidence that there is no persistent effect of ibuprofen exposure in former ELBW cases (Raaijmakers et al., 2018).

Observing a substantial increase of serum creatinine after birth for an ELBW neonate could reflect a pathological condition associated with an adverse drug reaction rather than the immaturity of the kidney. Along the same line, the absence of a decline in serum creatinine concentrations during the postnatal period might reflect impaired glomerular filtration and thus diminished kidney function. Although this study is not focusing on acute kidney injury, it needs to be acknowledged that physiological maturational events (i.e., adaptation to extrauterine life) early after birth cause serum creatinine concentrations to change, irrespective of (acute) kidney injury. Therefore, we emphasize incorporating annotations related to neonatal physiology in the definition of acute kidney injury. Furthermore, the recognition of drug toxicity signals in neonates, together with the extent of the adverse effect of ibuprofen, is also related to the postnatal age.

The small fluctuations that are being observed in creatinine concentrations and clearance can be identified as a limitation, albeit this study illustrates the impact of both maturational and non-maturational related effects, such as ibuprofen treatment on kidney function, which can help in recognizing an adverse drug reaction. In addition, the mechanism of action of ibuprofen is related to its nephrotoxic side-effects, making it challenging to disentangle the hemodynamically (“steal phenomenon”) and nephrotoxic effects (suppression prostaglandin synthesis) ibuprofen possesses. Both are related to aspects of renal perfusion.

We have captured the phenotypic pattern in the model, so we can only speculate the mechanistic contribution of either vascular steal or suppression of prostaglandin synthesis. The fact that ibuprofen clearance itself also increases with postnatal age, so that a maturational decrease in ibuprofen exposure may also be involved, further adds to the uncertainty of mechanisms involved (Engbers et al., 2020).

The implementation of measures to prevent or minimize nephrotoxicity and close monitoring of kidney functions are mandatory in the ELBW neonate treated with ibuprofen or other NSAIDs. Due to the substantial renal adverse drug reactions, it is essential to take into account the benefit-risk ratio when considering ibuprofen therapy (Bagnoli et al., 2013). The potential adverse drug reaction of ibuprofen affecting the kidney by a reduction of glomerular filtration is thought to depend on the degree of maturation of the neonate. Monitoring of serum creatinine concentrations, especially during the first week of life, is essential to early detect these maturational adverse drug reactions. After the first week of life, the effect can still be observed and quantified but is proportionally much more blunted (Figures 1, 2).

In conclusion, this study illustrates that the difference in serum creatinine concentrations decreases with postnatal age, indicating increased clearance capacity over time (gestational and postnatal age) and demonstrates the magnitude of the ibuprofen-related adverse drug reaction signals. Recognizing a change in creatinine concentrations might be useful in recognizing an adverse drug reaction signal after the start of ibuprofen treatment in ELBW neonates, but this signal is more pronounced in early neonatal life.

## Data Availability Statement

The raw data supporting the conclusions of this article will be made available by the authors, without undue reservation. Requests to access these data should be directed to karel.allegaert@uzleuven.be.

## Ethics Statement

The studies involving human participants were reviewed and approved by UZ Leuven, ec@uzleuven.be. Written informed consent from the participants’ legal guardians was not required to participate in this study in accordance with the national legislation and the institutional requirements.

## Author Contributions

TD, KA, and JA contributed to the conception and design of the study. TD performed the analysis and generated tables and figures. TD, KA, and JA wrote the first draft of the manuscript. KA, MP, AS, and JA contributed to the interpretation of the data and substantially revised the manuscript. All authors contributed to manuscript revision and approved the submitted version.

## Funding

TD, MP, and JA are funded by the Eckenstein-Geigy foundation. The research activities of AS are supported by the Clinical Research and Education Council of the University Hospitals Leuven.

## Conflict of Interest

The authors declare that the research was conducted in the absence of any commercial or financial relationships that could be construed as a potential conflict of interest.
